# Cervicovaginal fluid and semen block the microbicidal activity of hydrogen peroxide produced by vaginal lactobacilli

**DOI:** 10.1186/1471-2334-10-120

**Published:** 2010-05-19

**Authors:** Deirdre E O'Hanlon, Blair R Lanier, Thomas R Moench, Richard A Cone

**Affiliations:** 1 Mucosal Protection Laboratory, Thomas C. Jenkins Department of Biophysics, Johns Hopkins University, Baltimore, MD 21218, USA; 2 ReProtect, Inc., Baltimore, MD 21286, USA

## Abstract

**Background:**

H_2_O_2 _produced by vaginal lactobacilli is believed to protect against infection, and H_2_O_2_-producing lactobacilli inactivate pathogens *in vitro *in protein-free salt solution. However, cervicovaginal fluid (CVF) and semen have significant H_2_O_2_-blocking activity.

**Methods:**

We measured the H_2_O_2 _concentration of CVF and the H_2_O_2_-blocking activity of CVF and semen using fluorescence and *in vitro *bacterial-exposure experiments.

**Results:**

The mean H_2_O_2 _measured in fully aerobic CVF was 23 ± 5 μM; however, 50 μM H_2_O_2 _in salt solution showed no *in vitro *inactivation of HSV-2, *Neisseria gonorrhoeae*, *Hemophilus ducreyii*, or any of six BV-associated bacteria. CVF reduced 1 mM added H_2_O_2 _to an undetectable level, while semen reduced 10 mM added H_2_O_2 _to undetectable. Moreover, the addition of just 1% CVF supernatant abolished *in vitro *pathogen-inactivation by H_2_O_2_-producing lactobacilli.

**Conclusions:**

Given the H_2_O_2_-blocking activity of CVF and semen, it is implausible that H_2_O_2_-production by vaginal lactobacilli is a significant mechanism of protection *in vivo*.

## Background

The health of the female genital tract depends significantly upon the composition of the vaginal microflora. Bacterial vaginosis (BV) is a common microfloral disturbance: the lactobacilli that dominate a healthy vaginal microflora are replaced by a high-density, polymicrobial mix of other bacteria [1,2]. BV is associated with increased rates of many different genital tract infections, suggesting that vaginal lactobacilli provide broad-spectrum protection against pathogens.

Hydrogen peroxide is a broad-spectrum disinfectant, and cervicovaginal fluid (CVF) contains myeloperoxidase (MPO) that enhances pathogen-inactivation by H_2_O_2 _[3,4]. Epidemiological studies suggest that women with H_2_O_2_-producing lactobacilli are less likely to be infected with HIV-1, HSV-2, *Trichomonas vaginalis*, *Gardnerella vaginalis*, and gram-negative anaerobes associated with BV [5-7]. Several studies have reported that BV and H_2_O_2_-producing lactobacilli are strongly negatively associated: women with BV are between three and twelve times less likely to have H_2_O_2_-producing lactobacilli than women without BV [8-11].

Hydrogen peroxide-producing lactobacilli have also been shown to inactivate HIV-1 virions, and BV-associated bacteria when tested in protein-free salt solutions, giving support to the hypothesis that H_2_O_2_-production by vaginal lactobacilli is protective [12,13]. However, CVF and semen contain proteins, glycoproteins, polysaccharides, lipids, and other molecules with the potential to react with and inactivate H_2_O_2_. Additionally, the vagina is hypoxic most of the time, though the concentration of oxygen in the vagina increases following the insertion of a contraceptive diaphragm, during sexual arousal, and presumably during sexual intercourse [14-16]. Lactobacilli require oxygen to produce hydrogen peroxide: H_2_O_2 _concentration is undetectable during anaerobic culture, reaches 29-450 μM during aerobic culture, and 1.0-1.8 mM with vigorous aeration [17-19].

The primary aim of this study was to measure the H_2_O_2 _concentration of CVF from women with H_2_O_2_-producing lactobacilli microflora, and the H_2_O_2_-blocking activities of CVF and semen, to assess the likelihood that H_2_O_2 _produced by vaginal lactobacilli provides significant protection *in vivo*. Additionally, we tested whether H_2_O_2 _in simple salt solution at somewhat more than the concentration found in our CVF samples could inactivate vaginal pathogens, and whether CVF has the ability to block pathogen-inactivation by H_2_O_2_-producing lactobacilli.

## Methods

All materials and reagents were supplied by Sigma-Aldrich Inc. (St. Louis MO) unless otherwise specified; all microorganisms were supplied by the American Type Culture Collection (Manassas VA).

### Cervico-vaginal fluid and semen donors

The study was carried out at the Johns Hopkins University Homewood campus; participants were recruited primarily from among students and staff at the university. Our research conforms to the requirements of the Declaration of Helsinki, and the relevant federal and state laws; each participant gave written informed consent under a protocol approved by the Homewood Institutional Review Board on the Use of Human Subjects at Johns Hopkins University. Participants were required to be between 18 and 45 years old, and in good general health; female participants were at least three days past the most recent menstruation or unprotected penile-vaginal intercourse, at least three weeks past the most recent use of vaginal or systemic antimicrobials, and free from vaginal symptoms (discharge, odour, itching, or pain). Results from samples donated by six male and twenty-two female participants are reported here; the group comprised roughly equal numbers of non-Hispanic whites, blacks, and Asians, aged between 18 and 44 years old (mean age 26 ± 5 years).

### Collection of cervicovaginal fluid and semen samples

The non-absorbent disposable Instead^® ^menstrual cup (Instead Inc., La Jolla CA) was used to sample non-menstrual CVF. Unlike the more common collection methods of lavage, tampon, swab, or filter paper, the Instead^® ^cup collects a relatively large sample of CVF (a mix of cervical mucus, other secretions, and transudate) from a large area of the vagina without the use a speculum or dilution of the sample [20-22]. CVF adheres to the rim and both sides of the dome of the Instead^® ^cup, and is removed from the device by centrifugation.

The Instead^® ^cup was vaginally inserted, removed, and placed in a conical tube that was immediately transferred to a glove-box mimicking the hypoxia that generally prevails in the vagina: partial pressure of oxygen in the glove-box was 6.0 mm Hg ± 0.7 mm Hg, as measured with an MI-730 oxygen electrode (Microelectrodes Inc., Bedford NH), similar to the 4-14 mm Hg reported for the vagina [23]. Anaerotest^® ^indicator strips (manufacturer's estimated threshold of sensitivity 7 mm Hg, EMD Chemicals Inc., Gibbstown NJ) were used to ensure that the glove-box maintained hypoxic conditions throughout experiments. The tube containing the Instead^® ^was sparged with nitrogen and sealed. In all cases, the time elapsed between inserting the Instead^® ^into the vagina, sparging and sealing the tube in the glove-box was less than one minute. The sealed tube was removed from the glove-box, centrifuged for 1 minute at 1000 rpm (500*g*), and returned to the glove-box before being opened.

Semen samples were obtained by masturbation, maintained aerobically at room temperature, and used within 45 minutes of collection. Neither CVF nor semen samples were pooled; each experiment was performed several times (see results for *n *values) using samples from different donors.

### Evaluation of the samples

A smear from each sample of CVF was gram-stained and scored using the Nugent standardized system [24]. Samples for use in the study were restricted to those with Nugent scores ≤3 (indicating healthy vaginal microflora) and absence of vaginal leucorrhea (mean polymorphonuclear leukocyte per high powered field [PMNL/hpf] <10) [25]. A total of twenty-four female participants donated CVF; two samples were discarded due to Nugent scores >3, and no samples were excluded due to leucorrhea (mean PMNL/hpf of the included samples was 2.1). The low rate of discarded samples is consistent with the participant exclusion criteria (i.e., presence of any vaginal symptoms), and the study population's low-risk composition (generally young, affluent, with a high rate of condom use, and low rates of smoking, vaginal cleansing, and sexually transmitted infections).

H_2_O_2_-production in each CVF sample was assessed by growth on TMB-plus agar [26]; all samples met the criteria for H_2_O_2_-producing microflora [27], consistent with the characteristics of the study population [28,29]. An aliquot from each sample of semen was viability-stained, and the number of live sperm, dead sperm, and leukocytes present were scored [30]. All semen samples collected met WHO criteria for normal quality [31].

### Measuring hydrogen peroxide concentration in cervicovaginal fluid

Amplex Red^® ^(Invitrogen, Eugene OR) substrate in combination with horseradish peroxidase is a sensitive fluorescence assay for H_2_O_2 _[32]. Following the manufacturer's protocol, the assay reactions contained 50 μM Amplex Red^®^, 1 U/mL horseradish peroxidase, and 100 μL of CVF or semen in a 200 uL final reaction volume; however, the concentration of the reaction salt solution was increased from 50 mM to 250 mM to maintain an optimal reaction pH in the presence of CVF. Control experiments showed the change in salt solution concentration did not interfere with the ability of the assay to detect and quantify H_2_O_2 _(data not shown). The assay was carried out hypoxically or aerobically as indicated for the individual experiments. Control experiments showed that deoxygenating with nitrogen and incubating hypoxically did not interfere with the sensitivity or accuracy of the assay (data not shown).

### Measuring hydrogen peroxide-blocking activity of cervicovaginal fluid and semen

The oxidant-blocking activity in a sample differs with respect to different oxidant species [33,34]; we therefore tested the ability of CVF and semen to block exogenous H_2_O_2_. Aliquots of CVF or semen were diluted with an equal volume of 250 mM Na_2_HPO_4 _containing H_2_O_2 _at concentrations between 20 μM and 2 M, giving final H_2_O_2 _concentrations between 10 μM and 1 M. These aliquots were stirred for five seconds, and H_2_O_2 _concentration was measured aerobically using the Amplex Red^® ^assay.

### Measuring effect of aerobic exposure on hydrogen peroxide in cervicovaginal fluid

To estimate the amount of H_2_O_2 _produced in the generally hypoxic environment of the vagina, the H_2_O_2 _of CVF was measured immediately after sample collection and transfer to the hypoxic glove-box (i.e., after the approximately one minute aerobic exposure necessitated by the collection method). Some CVF samples were then maintained hypoxically at 37°C for one and a half hours, with an aliquot withdrawn every fifteen minutes and assayed hypoxically for H_2_O_2 _content.

To estimate the amount of H_2_O_2 _produced during periods of increased vaginal oxygen (as during sexual intercourse, when sexually transmitted pathogens might be introduced), CVF samples were first hypoxically equilibrated at 37°C for four hours, then exposed to air for one minute, fifteen minutes, or four hours and assayed for H_2_O_2 _content.

### Measuring effect of CVF supernatant on pathogen-inactivation by H_2_O_2_-producing lactobacilli

The production of H_2_O_2 _by *Lactobacillus crispatus *ATCC^® ^33820™ was confirmed by growth on TMB-plus agar. We replicated the protocol used by Klebanoff *et al *[35] to test *in vitro *pathogen-inactivation by H_2_O_2_-producing lactobacilli: *L. crispatus *was grown anaerobically in Difco™ Lactobacilli MRS broth (Becton, Dickinson and Co., Sparks MD) for approximately 24 hours; six hours before use in an experiment, the lactobacilli were transferred to peptone-yeast extract broth and grown aerobically with vigorous agitation. *Gardnerella vaginalis *ATCC^® ^14018™ was grown anaerobically in NYC III broth for approximately 24 hours; three hours before use in an experiment, the bacteria were transferred to peptone-starch-dextrose broth and grown aerobically with vigorous agitation. *Prevotella bivia *ATCC^® ^29303™ was grown anaerobically in chopped meat broth for approximately 24 hours. Immediately before an experiment, the cultures of all three bacterial species were washed twice in 100 mM Na_2_SO_4, _and re-suspended in Na_2_SO_4 _containing 100 mM NaCl and 48 mU/mL human MPO. A 250 μL aliquot of re-suspended *G. vaginalis *or *P. bivia *was mixed with an equal volume of re-suspended *L. crispatus*.

To avoid conflating endogenous vaginal bacteria with the cultured *L. crispatus*, *G. vaginalis*, and *P. bivia *used in these experiments, bacteria-depleted supernatants of CVF were prepared: each collected sample was diluted with a half-volume of 0.9% saline, mixed thoroughly, centrifuged at 1000 g for three minutes, and the supernatant drawn off for immediate use in an experiment [36]. Control experiments showed this centrifugation reduced bacterial concentrations in the diluted CVF from a mean of 5.6 × 10^7 ^cfu/mL to a mean of 4.0 × 10^1 ^cfu/mL (data not shown). The CVF supernatant (or an equal volume of saline as a negative control) was added to the washed and re-suspended *G. vaginalis *or *P. bivia *immediately before the addition of the *L. crispatus*. *G. vaginalis *mixtures were incubated aerobically at 37°C for 1 hour, then serially diluted with 100 mM Na_2_SO_4 _and plated onto blood agar that was incubated aerobically. *P. bivia *mixtures were incubated aerobically at 37°C for 30 minutes, then serially diluted with 0.9% saline containing 0.02% dithiothreitol, and duplicate-plated onto two sets of brucella agar plates; one set was incubated aerobically and the other anaerobically. After approximately 36 hours, plated colonies were counted: as in Klebanoff's experiments, *G. vaginalis *was distinguished from *L. crispatus *by colony morphology, growth habit, and presence of haemolysis on blood agar; *P. bivia *was distinguished from *L. crispatus *by colony morphology, growth habit, and the failure of *P. bivia *to grow on the aerobically incubated plates.

### Measuring pathogen-inactivation by 50 μM exogenous hydrogen peroxide

In these experiments, we replaced the H_2_O_2_-producing *L. crispatus *with exogenous H_2_O_2 _at a concentration above the concentration we measured in CVF samples, and expanded the list of target pathogens. *Mycoplasma hominis *ATCC^® ^14268™, *Mobiluncus curtsii *ATCC^® ^35241™, *Mobiluncus mulieris *ATCC^® ^35239™, *Peptostreptococcus anaerobius *ATCC^® ^27337™, *Hemophilus ducreyii *ATCC^® ^33940™, *G. vaginalis *and *P. bivia *were grown anaerobically, washed, and re-suspended as described for *P. bivia*, above. *Neisseria gonorrhoeae *ATCC^® ^19424™ was grown aerobically in ATCC^® ^medium #814, washed and re-suspended in the same way as *P. bivia*.

Aliquots of each individual bacterial culture were mixed with an equal volume of 100 mM Na_2_SO_4 _containing either 100 μM H_2_O_2 _with 100 mU/mL MPO, or 100 μM H_2_O_2 _without MPO, or plain salt solution as a negative control. The aliquots were incubated aerobically at 37°C for 1 hour, then serially diluted with 100 mM Na_2_SO_4 _and plated onto blood agar; plates were incubated for approximately 36 hours and colonies counted.

Aliquots of HSV-2 cell-free virus (ATCC^® ^VR-734™) were mixed with an equal volume of 100 μM H_2_O_2 _and 100 mU/mL MPO, or 100 μM H_2_O_2 _without MPO, or plain 100 mM Na_2_SO_4_, and incubated aerobically at 37°C for 30 minutes. The aliquots were then serially diluted with DMEM cell-growth medium supplemented with 10% fetal bovine serum (SAFC Biosciences, Lenexa KS) and plated onto ELVIS HSV-2 indicator cells (Diagnostic Hybrids, Athens OH), which were incubated, fixed, stained, and enumerated according to the manufacturer's instructions.

### Statistical analysis

Results are reported as means ± SDs of at least four independently repeated experiments. Difference between three or more means was tested using an ANOVA one-way analysis of variance; difference between two means was tested using a two-tailed Student's *t *test (comparisons are paired unless otherwise indicated in the results). Statistical analysis was performed using Microsoft Excel PHSTAT.

## Results

### Hydrogen peroxide in cervicovaginal fluid

**Figure 1:**
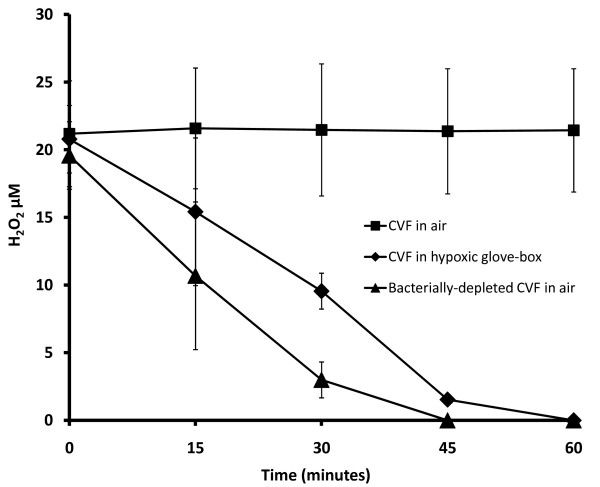
**Endogenous H_2_O_2 _concentration in cervico-vaginal fluid (CVF) measured by Amplex Red^® ^assay, as a function of time for: CVF in air, CVF in a hypoxic glove-box, and bacterially-depleted CVF in air; all samples were pre-exposed to air (*n *= 6 for each condition)**.

**Figure 2:**
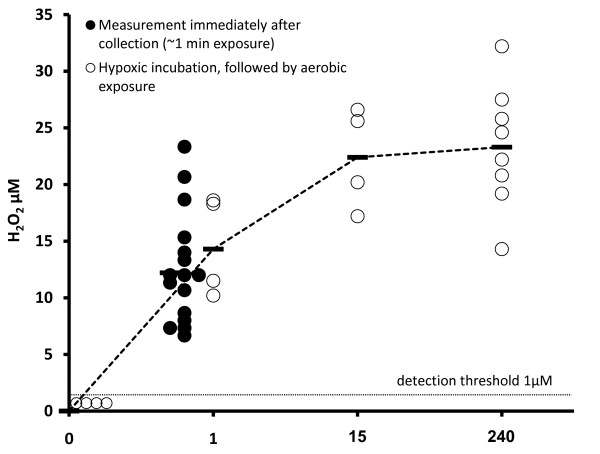
**Endogenous H_2_O_2 _concentration in CVF samples measured at 0, 1, 15 or 240 minutes exposure to air following four hours hypoxic incubation (open circles), or immediately after sample collection entailing ~1 minute exposure to air (closed circles, *n *= 16)**.

Hydrogen peroxide was only detectable after aerobic exposure: CVF samples (n = 6) incubated hypoxically for one hour had no detectable H_2_O_2 _remaining (Figure 1). Samples equilibrated hypoxically for four hours produced H_2_O_2 _following even brief exposures to air (Figure 2, open circles). H_2_O_2 _concentration was 15 μM ± 4 μM (n = 4) after 1 minute of aerobic exposure, and rose to 22 μM ± 4 μM after 15 minutes (n = 4), but H_2_O_2 _concentration did not continue to rise with further aerobic exposure. H_2_O_2 _concentration was 23 μM ± 5 μM after 4 hours in air (n = 8), not significantly increased (P = .36) compared to the concentration after 15 minutes (Figure 2, open circles).

The mean H_2_O_2 _content of CVF samples (n = 16) assayed immediately after sample collection and transfer to the hypoxic glove-box (i.e., after approximately one minute of aerobic exposure) was 12 μM ± 5 μM. There was no significant difference (unpaired comparison, *P *= .77) in H_2_O_2 _content between this group of CVF samples assayed immediately after collection, and the samples assayed after four hours hypoxic incubation followed by one minute of aerobic exposure (Figure 2, closed circles versus open circles), supporting our conclusion that 4 hours hypoxia had not affected the samples' ability to produce H_2_O_2 _when exposed to air.

### In vitro pathogen-inactivation by 50 μM exogenous hydrogen peroxide

Exogenous H_2_O_2 _at 50 μM (more than the maximum concentration we found in fully aerobically exposed CVF) had no effect on HSV-2, *N. gonorrhoeae*, *H. ducreyii*, or any of six BV-associated bacteria, when tested under *in vitro *conditions designed to maximize H_2_O_2 _pathogen-inactivation (i.e., with addition of MPO and NaCl, in a protein-free salt solution (Table 1).

**Table 1:** Viable organisms/mL before and after one hour exposure to 100 mM Na_2_SO_4_, 50 μM H_2_O_2_, or 50 μM H_2_O_2 _with 50 mU/mL MPO.

	Initial infectious *units/mL	Infectious *units/mL after 1 hour exposure			
			
		100 mM Na_2_SO_4_	50 μM H_2_O_2_	50 μM H_2_O_2 _with 50 mU/mL MPO	
HSV-2	1.0 × 10^5^	1.1 × 10^5^	1.2 × 10^5^	1.2 × 10^5^	NS (P = 0.45)

*N. gonorrhoeae*	1.5 × 10^7^	1.4 × 10^7^	1.3 × 10^7^	1.4 × 10^7^	NS (P = 0.79)

*H. ducreyi*	1.4 × 10^7^	1.1 × 10^7^	1.5 × 10^7^	1.4 × 10^7^	NS (P = 0.59)

*G. vaginalis*	8.2 × 10^7^	8.3 × 10^7^	8.3 × 10^7^	8.5 × 10^7^	NS (P = 0.55)

*P. bivia*	9.2 × 10^8^	9.0 × 10^5^	8.2 × 10^5^	8.8 × 10^5^	NS (P = 0.28)

*M. hominis*	7.8 × 10^7^	7.7 × 10^7^	7.6 × 10^7^	7.8 × 10^7^	NS (P = 0.39)

*M. curtsii*	6.1 × 10^7^	4.8 × 10^7^	4.2 × 10^7^	4.4 × 10^7^	NS (P = 0.22)

*M. mulieris*	8.5 × 10^7^	3.3 × 10^7^	3.4 × 10^7^	3.4 × 10^7^	NS (P = 0.19)

*P. anaerobius*	7.4 × 10^8^	4.0 × 10^6^	4.3 × 10^6^	4.4 × 10^6^	NS (P = 0.37)

P values from ANOVA comparison of post-exposure means for all three conditions.

* Colony-forming units/mL for bacteria, infected indicator cells/mL for HSV-2.

### Blocking of exogenous hydrogen peroxide by cervicovaginal fluid and semen

**Figure 3:**
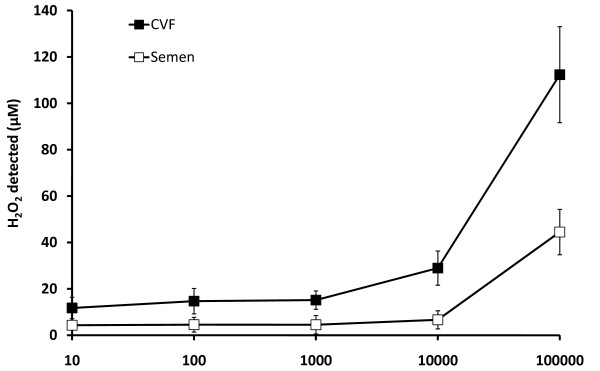
**H_2_O_2 _concentration detected by Amplex Red^® ^assay in CVF (*n *= 8) and semen (*n *= 6) samples, versus the concentration of exogenous H_2_O_2 _added to the samples**.

In all CVF samples tested (*n *= 8), addition of exogenous H_2_O_2 _to 1 mM produced no significant increase (*P *= .53) in the concentration of H_2_O_2 _detected (Figure 3, closed squares); in semen (*n *= 6), addition of H_2_O_2 _to 10 mM produced no significant increase (*P *= .72) (Figure 3, open squares).

### Blocking by CVF supernatant of pathogen-inactivation by H_2_O_2_-producing lactobacilli

**Figure 4:**
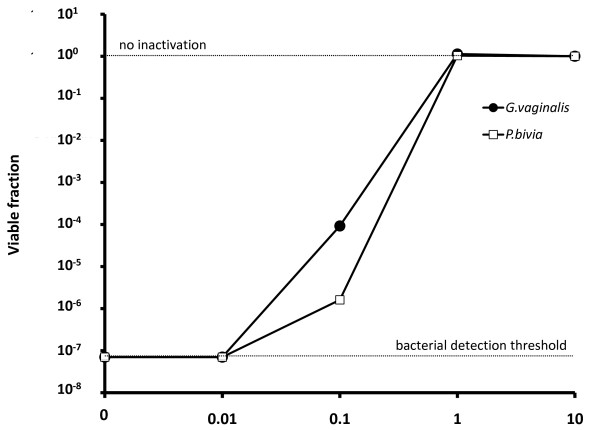
**Effect of CVF on the *in vitro *inactivation of *G. vaginalis *and *P. bivia *by H_2_O_2_-producing *L. crispatus***. Surviving fraction of organisms is plotted against concentration of CVF in the exposure solution.

Consistent with the report by Klebanoff *et al *of pathogen-inactivation by H_2_O_2_-producing lactobacilli, we found that *G. vaginalis *aerobically exposed for 1 hour to H_2_O_2_-producing *L. crispatus *in a salt solution containing MPO and NaCl showed complete inactivation; *P. bivia *exposed for 30 minutes to *L. crispatus *in salt solution containing MPO and NaCl also showed complete inactivation (Figure 4). However, the addition of CVF supernatant to a final CVF concentration of just 1% w/v abolished all inactivation; there was no significant reduction (paired comparisons, individual p values given in Table 1) in cfu/mL of *G. vaginalis *or *P. bivia *exposed for one hour to MPO, NaCl, 1% CVF, and H_2_O_2_-producing *L. crispatus*, compared to controls exposed to MPO, NaCl, 1% CVF, and no *L. crispatus*. As little as 0.1% CVF resulted in significant reduction in inactivation of *G. vaginalis *and *P. bivia *(*P *= 0.05).

## Discussion

Hydrogen peroxide production by vaginal lactobacilli has been widely emphasized as a mechanism of protection against genital tract pathogens. This view is based on epidemiological association, plausibility of mechanism, and *in vitro *studies of pathogen inactivation by H_2_O_2_-producing lactobacilli in protein-free salt solution, but CVF and semen contain proteins, glycoproteins, polysaccharides, lipids, and other molecules with H_2_O_2_-blocking activities. Indeed, a previous investigator noted that the toxicity of an *in vitro *H_2_O_2_-peroxidase-halide system against *Escherichia coli *was compromised by even trace amounts of protein [37]. Additionally, previous *in vitro *experiments were done under aerobic conditions that maximize H_2_O_2_-production, whereas the vagina is generally hypoxic.

Our hypoxic glove-box mimicked the hypoxic condition of the vagina. The H_2_O_2 _content of CVF samples decreased during incubation in the glove-box and became undetectable (<1 μM) within one hour, suggesting that H_2_O_2 _would generally be undetectable *in vivo*. The H_2_O_2 _of CVF samples collected with brief (~1 minute) aerobic exposure and assayed immediately under hypoxic conditions was not significantly different from that measured in samples equilibrated under hypoxic conditions (i.e., to the point where they contained no detectable H_2_O_2_) and then exposed to air for one minute. We conclude that the H_2_O_2 _detected in the CVF samples immediately following collection is attributable to the brief aerobic exposure.

The oxygen concentration in the vagina increases substantially during sexual intercourse. However, the mean H_2_O_2 _content in CVF samples after four hours of full exposure to air was still only 23 ± 5 μM, one hundred times lower than maximal aerobic *in vitro *production (~2 mM). We attribute this lower H_2_O_2 _content to the H_2_O_2_-blocking activity of CVF: even if H_2_O_2_-production reached millimolar concentrations, CVF had sufficient H_2_O_2_-blocking activity to leave only micromolar amounts available to the assay reaction (and presumably to pathogen-inactivating reactions *in vivo*). This interpretation is supported by our finding that CVF samples spiked with millimolar concentrations of exogenous H_2_O_2 _still only contained micromolar concentrations of detectable H_2_O_2_. It may seem surprising, given the copious H_2_O_2_-blocking activity of CVF, that any H_2_O_2 _could be detected at all. We attribute this to the continuing production of H_2_O_2 _by the lactobacilli in our CVF samples during and after aerobic exposure: when the Amplex Red^® ^substrate was added to the sample, it competed with the endogenous H_2_O_2_-blocking activity for the newly forming H_2_O_2_. Our hypothesis is supported by our finding that the H_2_O_2 _concentration decreased during aerobic incubation of bacterially-depleted CVF supernatant (n = 6), becoming undetectable after 45 minutes (Figure 1).

H_2_O_2 _at 50 μM (more than the maximum concentration we found in CVF) produced no inactivation of HSV-2, *N. gonorrhoeae*, *H. ducreyii*, or six major BV-associated bacteria, even in protein-free salt solution supplemented with MPO to enhance H_2_O_2 _toxicity. (*P. bivia *and *P. anaerobius *did show significant loss of viability (P = 0.01) irrespective of whether H_2_O_2 _was present or not, attributable to the effect of aerobic exposure on these strict anaerobes.) Additionally, the *in vitro *inactivation of *G. vaginalis *and *P. bivia *by H_2_O_2_-producing *L. crispatus *was completely abolished by the addition of only 1% CVF.

Our study has the following limitations: firstly, the concentration of H_2_O_2 _may be substantially higher in the immediate vicinity of H_2_O_2_-producing lactobacilli, and underestimated by our measurements in bulk CVF. A high local concentration of H_2_O_2 _might inactivate pathogens in close proximity to lactobacilli, even though the dispersed H_2_O_2 _concentration had no pathogen-inactivating activity (Table 1). Against this hypothesis is the fact that co-culture with H_2_O_2_-producing lactobacilli in the presence of CVF supernatant failed to inactivate both *G. vaginalis *and *P. bivia*, Second, the bacteria used in these experiments are established laboratory strains; primary isolates might be more sensitive to H_2_O_2_, although it is unclear how laboratory passage would provide selective pressure toward greater H_2_O_2 _resistance.

## Conclusions

Given the H_2_O_2_-blocking activity of CVF and semen, we conclude that H_2_O_2_-production by vaginal lactobacilli is implausible as a mechanism of direct protection against genital tract pathogens generally, and sexually-transmitted pathogens in particular. There remains the strong inverse association between H_2_O_2_-producing lactobacilli and BV. We hypothesize that lactobacilli strains that produce H_2_O_2 _may also produce more of other microbicidal factors such as lactic acid or bacteriocins, or alternatively, that H_2_O_2_-producing lactobacilli may be more susceptible to inhibitory factors elaborated by BV-associated organisms. We are currently investigating these possibilities.

## Competing interests

The authors declare that they have no competing interests.

## Authors' contributions

DEOH designed the study, collected data, analyzed the data, and prepared the manuscript. BL designed the study and collected data. TM participated in data analysis and the preparation of the manuscript. RC participated in data analysis and the preparation of the manuscript. All of the authors read and approved the final manuscript.

## Pre-publication history

The pre-publication history for this paper can be accessed here:

http://www.biomedcentral.com/1471-2334/10/120/prepub
